# Characterization of a Novel Caveolin Modulator That Reduces Vascular Permeability and Ocular Inflammation

**DOI:** 10.1167/tvst.10.6.21

**Published:** 2021-05-14

**Authors:** Pascal N. Bernatchez, Bo Tao, Ralph A. Bradshaw, David Eveleth, William C. Sessa

**Affiliations:** 1 Vascular Biology and Therapeutics Program and Department of Pharmacology, Yale University School of Medicine, New Haven, CT, USA; 2 CavtheRx, Hamden, CT, USA; 3 Trefoil Therapeutics, San Diego, CA, USA

**Keywords:** vascular permeability, vascular endothelial growth factor (VEGF), caveolin (Cav), peptides

## Abstract

**Purpose:**

Caveolin (Cav) regulates various aspect of endothelial cell signaling and cell-permeable peptides (CPPs) fused to domains of Cav can reduce retinal damage and inflammation in vivo. Thus, the goal of the present study was to identify a novel CPP that improves delivery of a truncated Cav modulator in vitro and in vivo.

**Methods:**

Phage display technology was used to identify a small peptide (RRPPR) that was internalized into endothelial cells. Fusions of Cav with the peptide were compared to existing molecules in three distinct assays, vascular endothelial growth factor-A (VEGF) induced nitric oxide (NO) release, VEGF induced vascular leakage, and in a model of immune mediated uveitis.

**Results:**

RRPPR was internalized efficiently and was potent in blocking NO release. Fusing RRPPR with a minimal Cav inhibitory domain (CVX51401) dose-dependently blocked NO release, VEGF induced permeability, and retinal damage in a model of uveitis.

**Conclusions:**

CVX51401 is a novel Cav modulator that reduces VEGF and immune mediated inflammation.

**Translational Relevance:**

CVX51401 is an optimized Cav modulator that reduces vascular permeability and ocular inflammation that is poised for clinical development.

## Introduction

The intracellular delivery of biologically active compounds that mimic or antagonize endogenous protein-protein interactions has proven to be very challenging because of the relative impermeability of the plasma membrane to polar compounds. Thus, the discovery of small cell-permeable peptides (CPPs) that serve as “Trojan horses” for carrying cargo molecules into living cells has generated considerable interest. Mounting evidence has demonstrated that CPP can allow efficient intracellular delivery of many molecules including oligonucleotides plasmids, viruses, peptides, and fluorophores^1^.^1^ The polybasic homeodomain of Antennapedia (AP; 16 amino acids), a Drosophila transcription factor, as well as the human immunodeficiency virus transactivator of transcription (TAT; 15 amino acids) are among the first CPPs described,^2^^,^^3^ and with common features of these peptides being amphipathic with a net positive charge.

The capacity of CPP to translocate cargo into cells makes them attractive delivery agents for cell-impermeable therapeutic compounds. By fusing the caveolin-1 (Cav) scaffolding domain to AP, we have shown that intracellular delivery of this peptide, called AP-Cav or cavtratin, attenuates inflammation in a variety of models.^4^^–^^6^ However, the efficacy, kinetics, safety profile, and specificity of CPP therapeutics in humans are unknown and it is reasonable to posit that a low molecular weight, targeted CPP with enhanced internalization capabilities may improve delivery to maximize the therapeutic activity in vivo.

Herein, we have isolated a very short five amino acid (RRPPR) CPP selected for its capacity to increase phage internalization into endothelial cells (ECs). Functional analyses reveal that RRPPR fused to an optimized domain of Cav (CVX51401) is more potent than AP-Cav at inhibiting vascular endothelial growth factor-A (VEGF) induced nitric oxide (NO) release in ECs, VEGF initiated vascular permeability in vivo and retinal degeneration in an immunological model of uveitis in mice. Thus, CVX51401 is a novel Cav modulator that may be a useful adjunct to reduce inflammation triggered by VEGF or immune activation.

## Methods

### Cell Isolation and Culture

Cultured rat heart microvascular EC (RHMVEC) were bought from VEC Technologies and grown on fibronectin coated plates in MCDB-131 complete medium (VEC Technologies). Bovine aortic ECs were grown in DMEM (high glucose; Cellgro) supplemented with 10% FBS (Hyclone) and pen/strep.

### T7 Phage Library Construction

Novagen T7 select phage display system was used for the random screening of peptides that facilitate EC uptake in conjunction with a pool of oligonucleotides randomly coding for 7-mer peptides. For amplification, the library was inoculated with BL21 culture (OD_600_ of 0.5–1.0) and induced with 1 mM IPTG at 37°C for 2 hours until cell lysis was observed. The lysate containing phages was clarified by centrifugation at 8000 g for 10 minutes, the supernatant was titered, and aliquots were stored a 4°C.

### Phage Selection by Uptake Into ECs

RHMVEC (80% confluent; approximately 2 × 10^7^ cells / 100 mm dish) were washed with PBS and pre-incubated in serum-free medium at 37°C for 30 minutes and inoculated with an extract (5 × 10^9^ pfu) of the T7 phage library to reach a multiplicity of infection (MOI) of 250. After incubation for 1 hour at 37°C, cells were washed with ice-cold PBS and acid washed with 0.1N HCl pH 2.2 for 15 seconds to remove unbounded and weakly associated phages from the cell surface. Cells were then trypsinized, centrifuged, and lysed with sterile deionized water on ice and internalized phages were amplified. After completion of 6 rounds of selection/amplification, *Escherichia Coli* BL21 was infected with the resulting phages and plated, individual plaques were picked, amplified, and sequenced. The peptide RRPPR was highly enriched and used in subsequent experiments.

### Peptide Synthesis

Peptides, corresponding to RQIKIWFQNRRMKWKK (*Antennapedia* CPP) or RRPPR with or without cargo fused to their C-terminus end (Cav-1 amino acids 82-101; DGIWKASFTTFTVTKYWFYR called cavtratin,^5^ RRPPR-Cav, or 82-95 DGIWKASFTTFTVT called CVX51401) were synthesized by standard Fmoc chemistry and analyzed by mass spectrometry to confirm purity by the W.M. Keck Biotechnology Resource Center at Yale University School of Medicine and CVX51401 synthesized by Bachem. All peptides were >99% pure. Prior to each experiment, desiccated peptides were weighed, dissolved in dimethyl sulfoxide (DMSO; J.T. Baker, Philipsburg, NJ) to 10 mM and diluted to 1 mM with distilled water. CVX 51401 was dissolved in ascorbic acid (20 mM) and mannitol (45 mg/mL) in distilled water.

### NO Release

VEGF-induced NO release experiments were performed as described.^6^ Briefly, confluent ECs were incubated in serum-free DMEM for 6 hours with peptides. Media was removed and fresh serum-free DMEM was added, with or without VEGF (1 nM) for 30 minutes. Media was collected, cells were trypsinized and counted, and nitrite levels in the supernatant were determined by using a Sievers NO chemiluminescence analyzer.

### Modified Miles Assay

Plasma protein leakage in mouse skin was studied using a modified Miles assay as described.^4^ Briefly, male C57/B6J mice (8–12 weeks old) were anesthetized with ketamine/xylazine and administered vehicle or CVX51401 (0.1, 1, 2.5, or 5 mg/Kg, IP) for 1 hour or to assess pharmacodynamics, a single dose of CVX51401 or vehicle was given from day 14 for 1 hour. Under these conditions, Evans Blue (30 mg/Kg; Sigma) was administered via the tail vein and allowed to circulate. The VEGF-A (300 ng) or PBS were injected intradermally (30 µl total) into the right and left dorsal ear skin, respectively. After 30 minutes, the mice were perfused with PBS, ears removed, blotted dry, and weighed. Evans blue was extracted with 500 µl of formamide for 24 hours at 55°C and measured spectrophotometrically at 610 nm.

### Mouse Model of Uveitis

Mice (B10.RIII) were immunized with the human interphotoreceptor retinoid binding protein (IRBP 1-20, 100 µg) peptide in 100 µl phosphate buffered saline emulsified in 100 µl of complete Freund's adjuvant to induce T cell mediated autoimmune uveoretinitis.^7^ Mice were then treated with vehicle, AP-Cav, or CVX51401 (at 5 mg/kg, IP) from days 7 to 14 postimmunization. At day 14, mice were euthanized and both eyes of each mouse processed for histopathology followed by disease grading by a reviewer masked to the treatment groups. The scoring system was: 0 = normal, 0.5 = trace and mild inflammatory cell infiltration with no tissue damage; 1 = infiltration with retinal folds and focal detachments, a few small granulomas in the choroid, retina with perivasculitis; 2 = moderate infiltration, with retinal folds, detachments, and focal photoreceptor damage with small to medium sized granulomas perivasculitis and vasculitis; 3 = medium to heavy infiltration, extensive retinal folding with detachments, and moderate photoreceptor damage, medium sized granulomatous lesions, and subretinal neovascularization; and 4 = heavy infiltration, diffuse retinal detachment with serous exudate and subretinal bleeding, extensive photoreceptor cell damage, large granulomatous lesions, and subretinal neovascularization.

### Statistical Analysis

Pairwise comparisons were made by an analysis of variance followed by a Dunnett's multiple comparison test. Data were considered significantly different if values of *P* < 0.05 were observed.

## Results

### Identification of a Novel Peptide That Promotes Internalization Into Endothelial Cells

A T7 phage display library that expressed, on average, 0.1 to 1 copy of randomly generated 7-mer peptides on the capsid was generated and a constant amount of input phages (5 × 10^9^) was added to cultured RHMVEC and phages selected for rapid cellular uptake within 20 minutes. After 6 rounds of infection/purification, a 100-fold increase in the percentage of recovered phages was observed. Following completion of biopanning and enrichment, the resulting phages were plated, and individual plaques were amplified and sequenced. Out of the 24 individual phages isolated, we observe that 5 coded for the unexpectedly short 5-mer peptide, RRPPR, as the most frequently identified peptide.

### RRPPR Is Internalized More Efficiently Than AP in ECs

**Figure 1.**
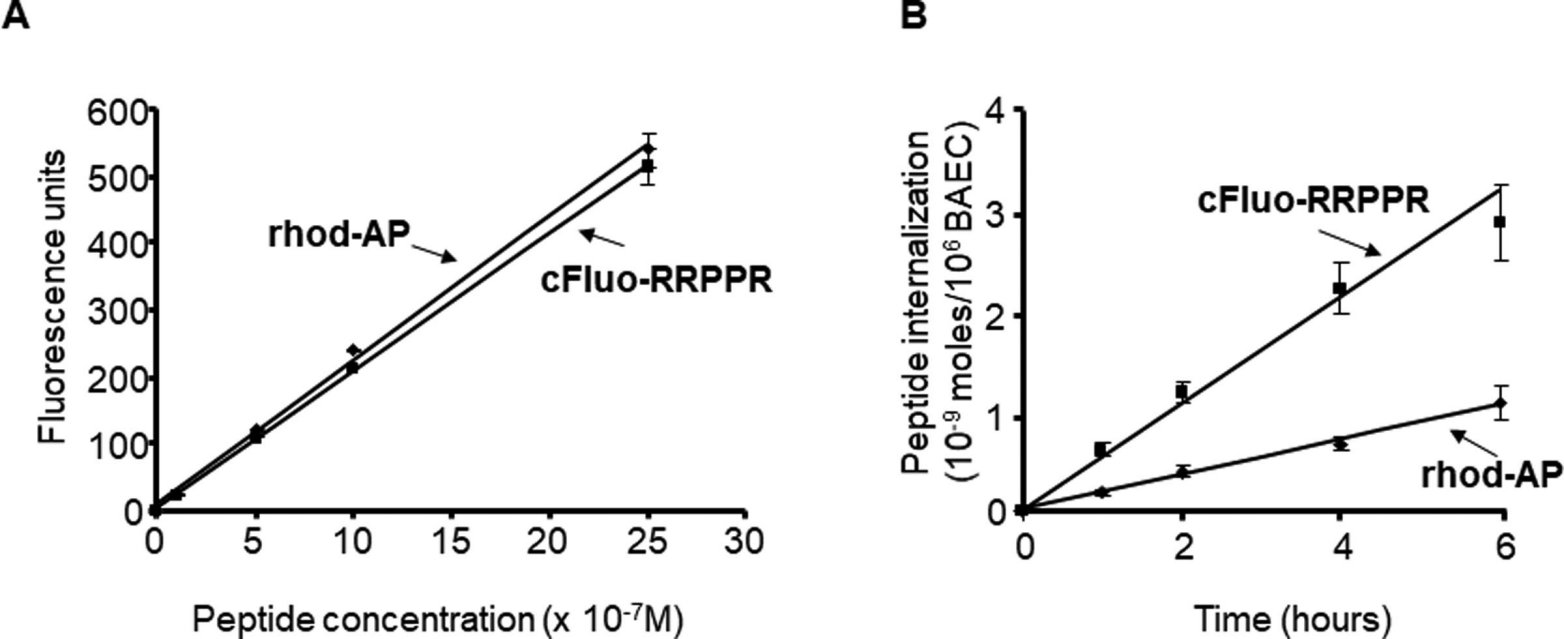
**RRPPR intern** **a** **lizes at a faster rate and more completely than AP in cultured EC.** (**A**) Comparative fluorescence readouts for similar concentrations of rhodamine-AP (rhod-AP) and carboxyfluorescein-Endo5 (cFluo-RRPPR) dissolved in the cell lysis buffer to assess the linearity between peptide concentration and fluorescence values. Peptides were assayed separately to prevent interference of absorption/emission spectra. (**B**) Rate of cFluo-RRPPR internalization is greater than that of rhod-AP. Cultured ECs were incubated for 1, 2, 4, or 6 hours with individual peptides, acid washed, rinsed, trypsinized, lysed, and fluorescence was determined and converted to moles of peptides per 10^6^ cells by using a standard curve. Cells incubated with peptides for 5 minutes and treated as described, were used as background values. Data are mean ± standard error of the mean (SEM). *N* = 4 wells per condition, in triplicate, representative data are shown.

AP is a commonly used CPP, thus, we directly compared the internalization rate of RRPPR to that of AP by using carboxyfluorescein (cFluo) - and rhodamine (rhod) - labeled forms of each peptide, respectively (termed cFluo-RRPPR and rhod-AP). First, we confirmed the linearity of each fluorophore-coupled peptide by performing a standard concentration fluorescence curve. As shown in Figure 1A, calibration was performed to obtain similar absorbance values for both fluorophores (by adjusting gain settings for each fluorophores). Next, ECs were incubated separately with fluorophore-labeled peptides (10^−6^ M) for 1, 2, 4, and 6 hours, acid washed, lysed, and total peptide uptake determined by quantifying cellular fluorescence. There was a linear increase in rhod-AP internalization over time (Fig. 1B) peaking at 6 hours at a value of 1.07 × 10^−9^ moles of rhod-AP/10^6^ cells, which represented approximately 11% of the total amount of added rhod-AP at time 0. Interestingly, the rate and extent of internalization of cFluo-RRPPR is augmented relative to AP, also peaking at 6 hours with a value of 2.85 × 10^−9^ moles of cFluo-RRPPR/10^6^ cells, indicating that 30.5% of the initial peptide was internalized after 6 hours.

### RRPPR-Cav Shows Greater Endothelial Nitric Oxide Synthase Inhibitory Activity Than AP-Cav

**Figure 2.**
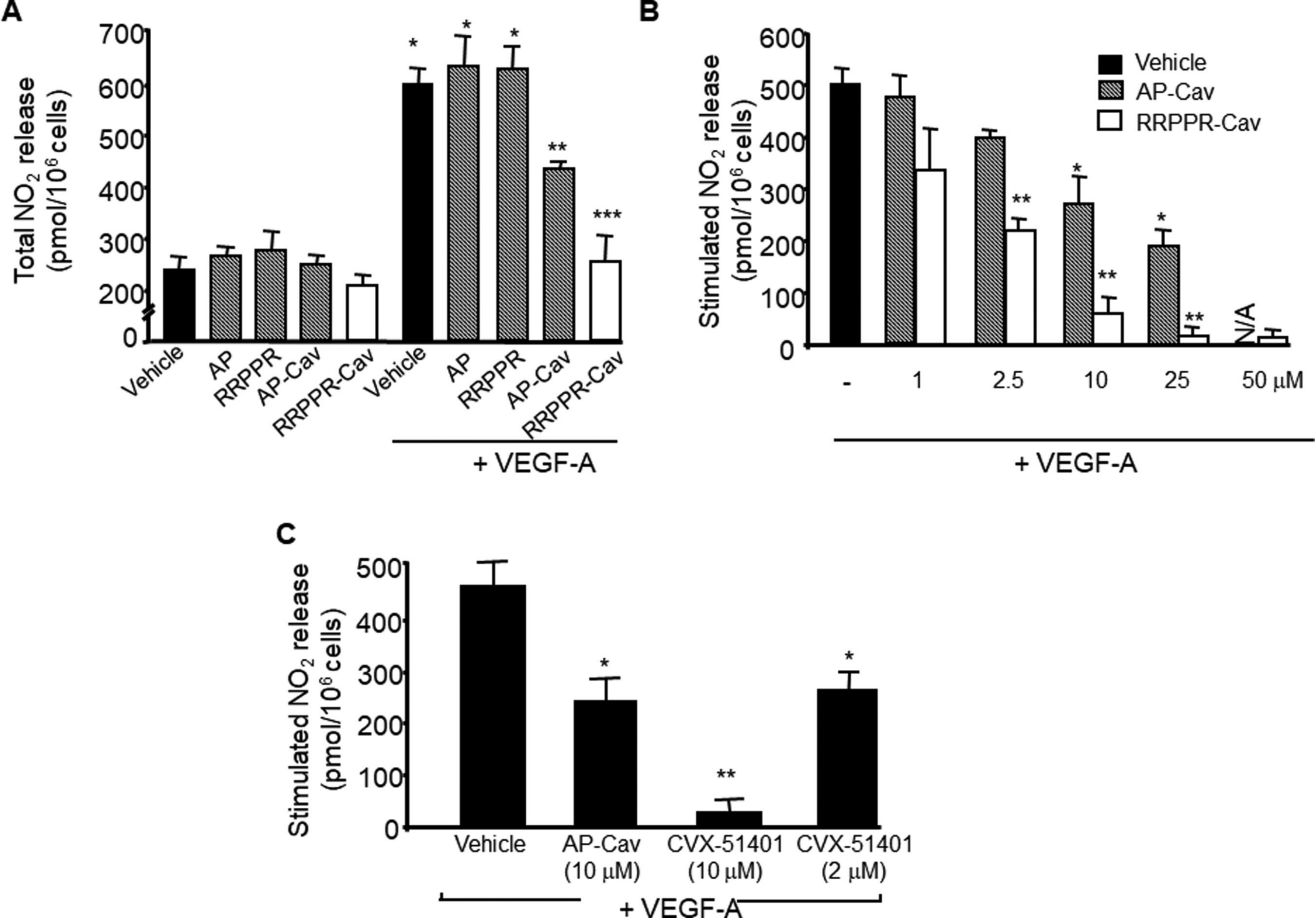
**RRPPR-Cav is more potent than AP-Cav at blocking VEGF-induced NO release.** (**A**) Cultured ECs were pretreated for 6 hours with the indicated peptides (10 µM) and stimulated with VEGF (1 nM) for 30 minutes as indicated. *P < 0.05 compared with vehicle, and **P < 0.05 compared with AP-Cav + VEGF. *N* = 4 in triplicate. (**B**) The inhibitory actions of AP-Cav and RRPPR-Cav on NO release are dose-dependent. Cultured ECs were pretreated with peptides (1–50 µM) for 6 hours and stimulated with VEGF as described in **A**. Data are mean+/− SEM with *P < 0.05 compared with vehicle, and **P < 0.05 compared with AP-Cav + VEGF. *N* = 4 experiments performed in duplicate. (**C**) Optimization of both the cell-penetrating sequence and Cav domain (CVX51401) results in more effective NO release inhibition. ECs were treated with AP-Cav (10 µM) or CVX51401 (2 and 10 µM) and VEGF induced NO release assessed. Data are mean +/− SEM with *P < 0.05 compared with vehicle, and **P < 0.05 compared with AP-Cav (10 µM). *N* = 4 experiments were performed in triplicate, representative data are shown.

We have and others have previously described the capacity of a synthetic, AP amino terminal Cav scaffolding domain fusion called AP-Cav (i.e.cavtratin^5^) to block agonist-induced endothelial nitric oxide synthase (eNOS) activity and NO releasee from cultured ECs^4^^,^^6^ as a bioassay for peptide function. Thus, we compared the potential of RRPPR-Cav with that of AP-Cav by testing their effects on basal and VEGF induced NO release from cultured bovine aortic ECs. Treatment of cells with AP, RRPPR, AP-Cav, or RRPPR-Cav (10 µM of each) did not impact basal NO release; and the control peptides, AP, and RRPPR, did not impact VEGF induced NO release. However, AP-Cav and RRPPR-Cav, blunted VEGF induced NO release from EC (Fig. 2A). The effects of the peptides were dose-dependent, with RRPPR-Cav being more potent than AP-Cav (Fig. 2B; half-maximal effective concentration [EC_50_] of RRPPR-Cav and AP-Cav were 1.8 × 10^−6^ and 7.5 × 10^−6^ M, respectively).

Previous work has shown that amino acids 82-95 of Cav-1 is the smallest pharmacophore that mediates the inhibitory action on eNOS in vitro and in two models of acute inflammation in vivo*.*^6^ Therefore, we synthesized RRPPR on the N terminus of Cav 82-95 (RRPPR-Cav 82-95, which is 19 amino acids in length; called CVX51401) to test its efficacy in vitro and in vivo. Pretreatment of BAEC with CVX51401 (10 µM) markedly reduced VEGF-induced NO release, whereas an identical concentration of AP-Cav blocked NO release to a lesser extent (Fig. 2C) and treatment with a lower concentration of CVX51401 (2 µM) had a similar effect to AP-Cav (10 µM).

### CVX51401 Shows Anti-Permeability Properties and a Sustained Pharmacodynamic Response In Vivo

**Figure 3.**
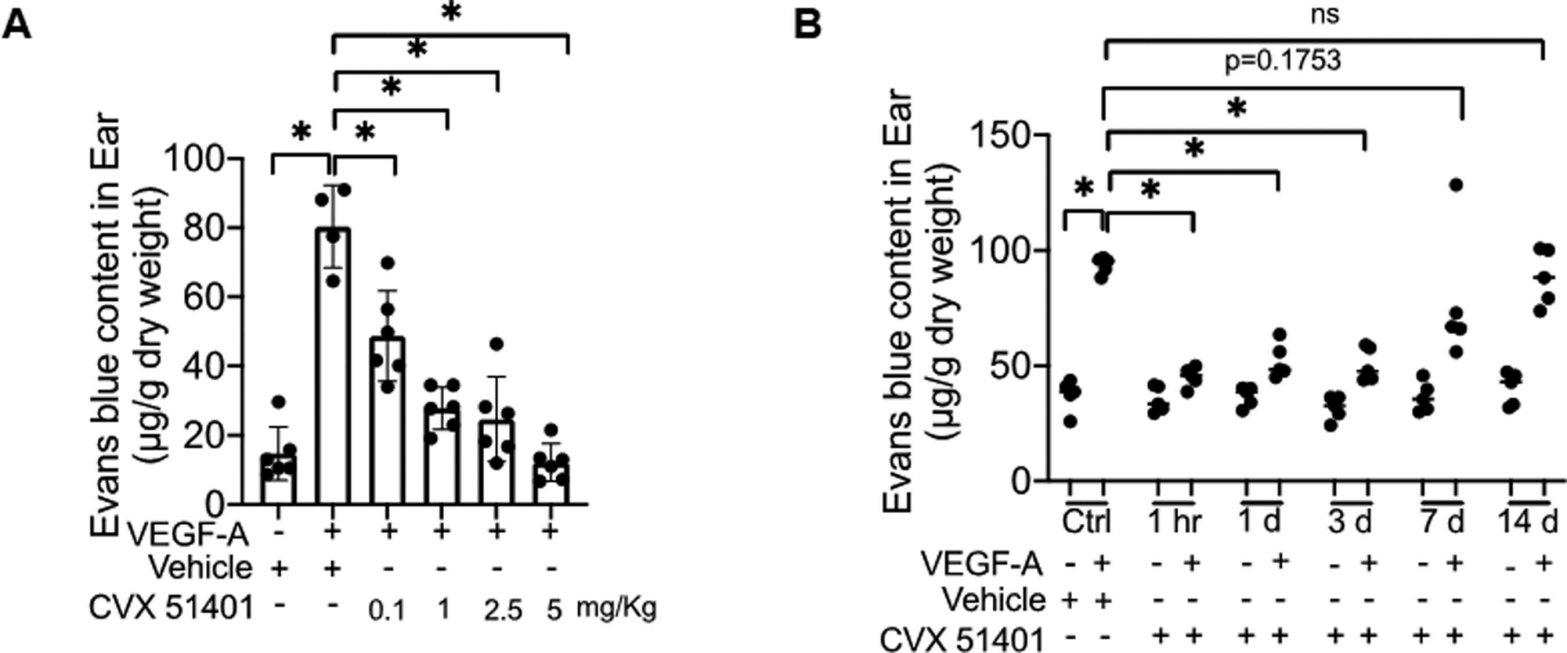
**CVX51401 suppresses VEGF-A induced permeability in vivo.** (**A**) C57/B6J mice were administrated of either vehicle or CVX 51401 (0.1–5 mg/kg, IP) for 1 hour; followed by VEGF-A (300 ng)or PBS injected intradermally into either ear for 30 minutes and the extravasation of Evans Blue measured spectrophotometrically (*n* = 4–6 mice/group). Data are mean +/− standard error of measurement with *P < 0.05 compared with vehicle. (**B**) Mice were injected on days 14, 7, 3, and 1 and after 1 hour, with a single dose of CVX 51401 (2.5 mg/kg; IP) or vehicle. All 6 groups of mice were then injected intradermally into the ear with either PBS or VEGF-A and Evans blue leakage quantified. Data are mean +/− standard error of measurement with data are mean ± SEM. *P < 0.05 compared with vehicle and PBS injected in the ears (*n* = 5 mice/group).

VEGF and several other bioactive molecules can promote NO signaling, vascular leakage, and inflammation, and these effects can be reduced by administration of cavtratin.^4^^-^^6^^,^^8^^,^^9^ To test the efficacy of CVX51401 on vascular permeability, mice were treated with CVX51401 (0.1–5 mg/kg, IP) for 20 to 30 minutes and VEGF induced permeability of the skin examined using Evans Blue dye extravasation as an index of vascular protein leakage. As seen in Figure 3A, VEGF induced a marked increase in Evans blue extravasation and CVX51401 dose-dependently reduced vascular permeability in vivo. Next, we tested the pharmacodynamic actions of a single dose of CVX51401 on VEGF induced vascular leakage. Mice were administered CVX51401 (2.5 mg/kg; IP) and at several time points after administration (1 hour followed by 1–14 days), VEGF induced vascular permeability of the skin examined. As seen in Figure 3B, under control conditions, VEGF induced vascular leakage that was largely blocked by an hour pretreatment with CVX51401. Remarkably, a single dose of the peptide exerted a sustained anti-inflammatory effect that lasted at least 3 days and waned between day 3 and 7, and by day 14 the inhibitory actions of CVX51401 were no longer present.

### CVX51401 Reduces Retinal Damage in a Model of Murine Uveitis

**Figure 4.**
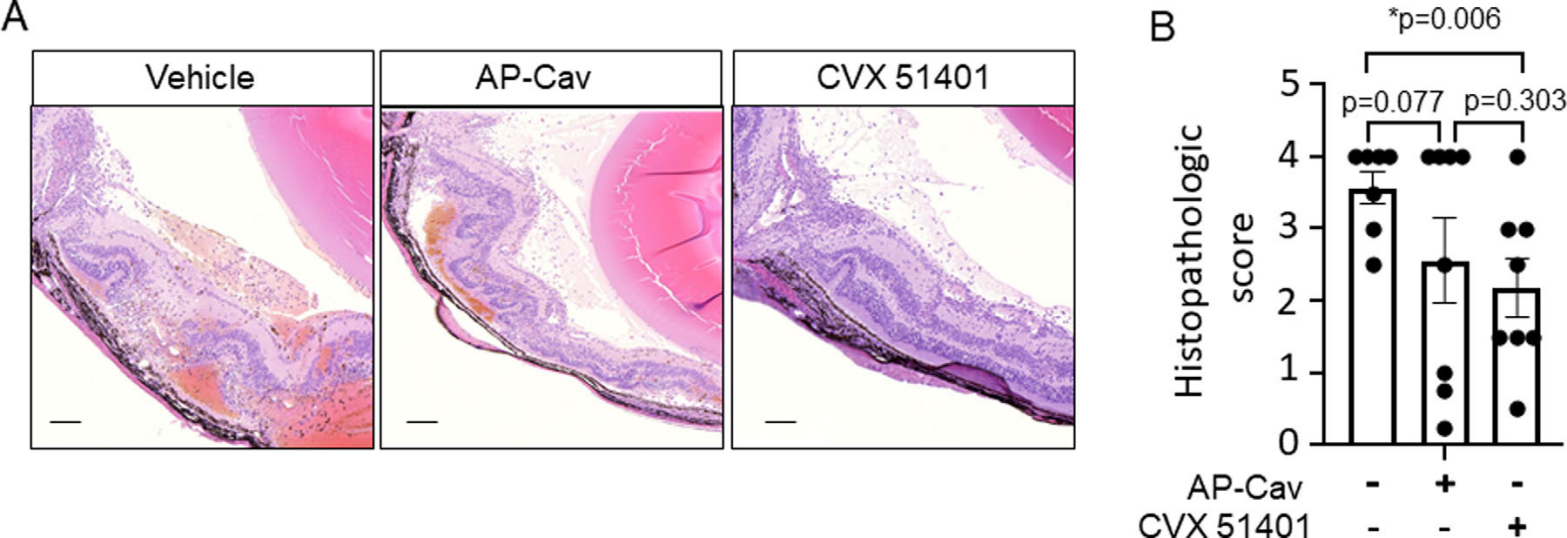
**CVX51401 reduces inflammation and retinal degeneration in a model of uveitis in mice.** B10.RIII mice were immunized with IRBP in complete adjuvant and treated with vehicle, AP-Cav, or CVX51401 (at 5 mg/kg daily, IP) from days 7 to 14 post-immunization. At day 14, eyes were processed for histology followed by grading. (**A**) Representative retinal histologically of treated groups showing less inflammation and degeneration in CVX51401 treated mice and quantitative scoring of these results in (**B**). Data are mean +/− standard error of measurement with *P < 0.05 compared with vehicle (*n* = 7 mice), AP-Cav (*n* = 8 mice), and CVX51401 (*n* = 8 mice). *P* values for other groups are shown for comparison.

Because Cav-1 and Cav modulators may impact nonimmune and immune mediated inflammation of the eyes, we examined the effects of AP-Cav and CVX51401 in a murine model of immune uveitis.^7^ Immunizing B10.RIII mice with human IRBP peptide, induces T cell mediated retinal degeneration as a hallmark of immune uveitis. Thus, mice were immunized with human IRBP peptide and treated therapeutically with vehicle, AP-Cav, or CVX51401 (5 mg/kg; IP daily from days 7–14 post-immunization). CVX51401, but not AP-Cav, reduced histopathological uveitis score when administered during the development of uveitis (Figs. 4A, 4B for quantitation). However, there was no statistical difference between AP-Cav versus CVX51401 treated groups.

## Discussion

In this paper, we describe a new, short CPP that improves cellular uptake into ECs and when synthesized with the inhibitory domain of Cav, exerts a more potent action than AP-Cav. In addition, minimizing the active domain of Cav in conjunction with RRPPR as the CPP led to more potent inhibitory effect that reduced VEGF stimulated NO release, VEGF induced edema, and retinal degeneration in a model of immune uveitis. Thus, CVX51401 is an improved Cav-1 modulator that may be a useful adjunct to treat disorders associated with heightened vascular permeability and inflammation.

Previous work has shown that AP-Cav, called cavtratin,^5^ has beneficial therapeutic effects in a variety of models, including pulmonary fibrosis,^10^ hypertension,^11^ hyperoxic lung damage,^12^ heart failure,^13^ sepsis,^14^^,^^15^ liver fibrosis,^16^ cancer,^5^ multiple sclerosis (MS),^17^ asthma,^18^ and ocular diseases, including ischemic and VEGF driven retinal degeneration.^19^ Although these models reflect distinct pathological features in different organs, there are common features in the models of enhanced vascular permeability, inflammation, and macrophage recruitment and polarization. The precise molecular target(s) of AP-Cav are not known and may be elusive due to the pleiotropic nature of the Cavs ability to potentially interact with different proteins. Moreover, the interactions of AP-Cav may be stimulatory or inhibitory depending on the affected protein or may impact the lipid composition of cells.^20^ In all the above studies with AP-Cav as a tool, the intracellular delivery of the peptide is critical for the effects because the targets of Cav are all intracellular. Recently, a novel Cav therapeutic for the treatment of idiopathic pulmonary fibrosis (IPF) has been developed based on the delivery of small fragment of the Cav scaffolding domain (7 amino acids from 89–95 called CSP7) that is effective in models of pulmonary fibrosis.^21^ Interestingly, this domain is encompassed in AP-Cav, as well as CVX51401. Remarkably, this naked peptide has a profound effect in reducing the profibrotic TGF-β signaling pathway in cells and in vivo and a modified formulation has been developed for the administration of the peptide in patients with IPF. The effectiveness of CSP7 implies that epithelial cells may have the machinery to internalize the peptide in the absence of a CPP attached. These exciting observations implore the clinical development of other Cav modulators, such as CVX51401 for additional indications.

Previous work by our group has documented the importance of eNOS as a downstream regulator of VEGF signaling^5^^,^^6^^,^^22^ and that Cav-1 serves as a negative regulator of eNOS by virtue of the Cav scaffolding domain directly inhibiting eNOS.^23^^–^^25^ Physiologically, VEGF induced changes in vascular permeability are mediated, in part, by NO because eNOS deficient mice are resistant to VEGF induced changes in vascular permeability.^9^^,^^26^^,^^27^ Thus, a major bioassay used for the development of AP-Cav and its derivatives were screened against VEGF induced NO release from EC and VEGF driven edema in vivo. Indeed, in the present study, RRPPR-Cav and CVX51401 strongly blunted VEGF induced NO release compared to AP-Cav and CVX51401 reduced VEGF driven changes in vascular permeability of the skin. It would be of interest to test CVX51401 in models of retinal and choroidal leakage and neovascularization because AP-Cav was additive with an anti-VEGF antibody in reducing retinal disease and more recently shown to reduce epithelial-mesenchymal transition and laser induced subretinal fibrosis.^28^

Uveitis is a collection of conditions causing inflammation of the uveal tract (iris, ciliary body, and choroid) and often involves the retina. Current treatment goals are the suppression of inflammation using steroids in combination with immunomodulators such as methotrexate, mycophenolate mofetil, and biologic modifiers in combination.^29^ Key components of the inflammatory process in uveitis include the invasion of the eye by both T cells and macrophages, breakdown of the blood-retinal barrier, edema, and leakage of protein into the aqueous and vitreous. Prior work has shown that AP-Cav reduced the influx of inflammatory cells in an immune models of MS, asthma, and retinal disease^17^^–^^19^^,^^28^ and reduced the actions and production of cytokines.^15^^,^^30^ Therefore, we examined the actions of CVX51401 in the IRBP model of uveitis that has a strong immunological and cytokine component to disease pathogenesis. Systemic administration of CVX51401 in a therapeutic dosing regimen reduced indices of inflammation and retinal degeneration. Mechanistically, it is feasible that CVX51401 inhibits macrophage or microglial migration and induces JNK activation^19^ or inhibits different NOS isoforms (eNOS and inducible NOS^23^) reducing disease pathogenesis. Thus, CVX51401 may be a useful adjunctive therapeutic compound for retinal disease associated with heightened vascular permeability, sustained inflammation, and fibrosis.

In summary, CVX51401 is a next generation Cav modulator optimized to improve cellular uptake and efficacy with a pharmacodynamic effect of a single dose lasting at least 3 days. Additional optimization to determine the route of administration, pharmacokinetic, and pharmacodynamic profiles of CVX51401 are critical for developing the therapeutic potential necessary for diseases that require chronic dosing, such as macular degeneration and uveitis.
